# A large receptive–expressive gap in bilingual children

**DOI:** 10.3389/fpsyg.2015.01284

**Published:** 2015-08-25

**Authors:** Karin Keller, Larissa M. Troesch, Alexander Grob

**Affiliations:** ^1^Department of Psychology and Human Development, Institute of Education, University College LondonLondon, UK; ^2^Department of Psychology, University of BaselBasel, Switzerland

**Keywords:** receptive language, expressive language, second language, bilingualism, receptive–expressive gap, language exposure

## Abstract

The present study focuses on the discrepancy between receptive and expressive language competence among bilingual children and tests possible explanatory factors of this gap. The sample consisted of 406 bilingual children with German as their second language (L2) and 46 different first languages. Receptive and expressive German language competence (L2) were measured with a standardized language development test at the age of 43 months. As expected, a significant gap in receptive and expressive German language competence (L2) emerged in all language groups. The size of the gap reached 1 SD and correlated with the amount of language contact and thus provides support for the language exposure hypothesis. However, we found no evidence for the language familiarity hypothesis. The present study contributes to the understanding of mechanisms in bilingual language development and, hence, is consequential for both basic research and language assessment practice.

## Introduction

Growing up bilingual is related to a series of advantages as well as disadvantages. In some cognitive and linguistic development domains bilingual children have an advantage over their monolingual peers (see Barac et al., 2014 for an overview). Bilingual compared to monolingual children show higher test scores in tasks on cognitive flexibility (e.g., Chen et al., 2014), memory (e.g., Brito et al., 2014), attention (e.g., Bialystok, 2015), control of interference (e.g., Filippi et al., 2015), metalinguistic, and language pragmatic competence (e.g., Kang, 2012) and visual language discrimination (e.g., Sebastian-Galles et al., 2012); however, in other development domains, such as vocabulary in the first (L1) and the second language (L2), bilingual children lag behind their monolingual peers (e.g., Pearson et al., 1993; Farnia and Geva, 2011; Hoff, 2013). This specifically bilingual pattern of competence not only is evident in the different cognitive and linguistic development domains, but also exists between the two modalities, that is, language comprehension and language production. Thus bilingual individuals exhibit a more pronounced discrepancy between receptive and expressive language competence as compared to monolingual individuals (e.g., Yan and Nicoladis, 2009; Gibson et al., 2012).

The present study examines the central characteristics and explanatory approaches of this so-called *receptive–expressive gap* in L2. Regarding the explanatory approaches, we firstly examine whether the extent of the gap in L2 is due to the *language familiarity* of the first and second language. Second, we investigate whether the amount of contact with speakers of the second language—i.e., *language exposure*—could be a cause of the difference.

### Characteristics of the Receptive–Expressive Gap

The receptive–expressive gap needs to be distinguished from the more widely known normative phenomenon of higher receptive versus expressive language competence. It represents an unexpected discrepancy between receptive and expressive language that goes beyond the normal asymmetry between the two modalities (cf. Gibson et al., 2014). In order to distinguish a receptive–expressive gap from normative modality asymmetries, standard scores of language tests are usually employed. Standard scores are transformed raw scores of the norm sample that were trimmed to a standard normal distribution within the procedure of test construction. Accordingly, the subscales of a test have the same mean and the same SD, regardless of the difficulty of the respective task. The most common standard scores are IQ values (*M* = 100, SD = 15), *T*-values (*M* = 50, SD = 10), and *z*-values (*M* = 0, SD = 1). A receptive–expressive gap is defined by a difference between the standard scores of the receptive and expressive tasks in the test. On a group level a receptive–expressive gap is defined by a significant difference between the two means, on an individual level by a difference of at least two thirds of a SD (Gibson et al., 2014).

The receptive–expressive gap of bilingual children has hitherto rarely been examined and tested for statistical significance (Gibson et al., 2012), although a series of studies indicates that bilingual children experience more difficulties in language production in comparison to language comprehension (Pearson et al., 1993; Grüter, 2005; Swanson et al., 2008; Sachse et al., 2010). In general, the gap is manifested in both L1 and L2, but in the case of immigrant children it is more pronounced in L1—i.e., the native language. In L1 a difference of one or more than 1 SD is often reported, whereas in L2—which is the focus of the current study—a clearly smaller gap of maximum one-half a SD (Hutchinson et al., 2003; Windsor and Kohnert, 2004; Swanson et al., 2008; Gibson et al., 2012) or no gap is reported (Kan and Kohnert, 2005; Barnett et al., 2007; Oller et al., 2007; Lesaux et al., 2010). Gibson et al.’s (2014) study represents an exception in regard to this gap pattern. Here, children with English as a second language had no gap in L1, but instead had a markedly larger gap of 0.3–1.3 SD in L2.

The gap has been recognized among various L1s and L2s; for example, among Spanish–English speaking children (Windsor and Kohnert, 2004; Oller et al., 2007; Swanson et al., 2008; Gibson et al., 2012, 2014), French–English (Yan and Nicoladis, 2009), Dutch–Moroccan (Verhoeven, 2000), Hwang–English (Kan and Kohnert, 2005), Samoan–English (Hemsley et al., 2010), and Mandarin–English speaking children (Sheng et al., 2011), and in a culturally diverse group of children with German as L2 (Sachse et al., 2010). These studies lead to the conclusion that the gap is not limited to a specific language.

### Possible Causes of the Gap

Even though a large number of studies on bilingual children have indicated a difference between language comprehension and language production, the causes of the receptive–expressive gap are largely unexplored (Gibson et al., 2012). Furthermore, it is unclear whether the reasons for the gaps in L1 and L2 among bilingual preschool children are similar, or whether different processes are at work. Literature on the L2 gap, which is the focus of the present study, debates the language familiarity hypothesis and the language exposure hypothesis in particular. Both explanatory approaches focus on different processes and are thus not mutually exclusive.

The *language familiarity hypothesis* refers to the interdependence hypothesis (Cummins, 1979), which assumes that L1 and L2 knowledge can be mutually supportive (Oller and Jarmulowicz, 2007). Research is divided over which elements are transferred in which way and whether a transfer can already be assumed at preschool age (cf. Genesee and Geva, 2006). However, it is assumed that the transfer potential is higher for linguistically related languages. Furthermore, it is assumed in connection with the receptive–expressive gap that the transfer works differently for the two language modalities (Oller and Jarmulowicz, 2007) and that the transfer potential from L1 to L2 lies—at least in the domain of vocabulary—particularly in language comprehension and is considered to be slighter for language production. According to the language familiarity hypothesis, children with two linguistically related languages (e.g., English–German) show a greater receptive–expressive gap, as the transfer is greater in the receptive area and is favored by the close familiarity of the two languages. In contrast, among children with two linguistically unrelated languages (e.g., Tamil–German) a smaller gap is expected given the more restricted possibilities for transfers.

Whether language familiarity is linked to the size of the gap has not yet been examined empirically. Current literature assumes a gap in the different languages and with different language familiarities (Verhoeven, 2000; Windsor and Kohnert, 2004; Kan and Kohnert, 2005; Oller et al., 2007; Swanson et al., 2008; Yan and Nicoladis, 2009; Hemsley et al., 2010; Sachse et al., 2010; Sheng et al., 2011; Gibson et al., 2012, 2014), but it is not possible to compare the extent of these gaps across different studies because of the different sample characteristics and different measures. In the present study we examine whether the gap size varies between groups of children with different levels of familiarity of L1 and L2 (e.g., Tamil–German vs. Spanish–German vs. English–German etc.).

The *language exposure hypothesis*, also advocated by Oller et al. (2007), traces the causes of the gap back to the degree of language exposure. Language input is distributed over two languages in bilingual children resulting in less input for each of the languages in comparison to the input among monolingual peers (Pearson et al., 1993). However, bilinguals not only experience less linguistic input but also have less opportunity to speak the respective language. Gollan et al. (2002) assumed that less language practice is attended by a weaker connection between semantic and lexical structures (weaker links hypothesis), which hampers the lexical access that is essential for language production (Gollan and Acenas, 2004; Yan and Nicoladis, 2009).

This weak link hinders language production but has little or no effect on language comprehension, which results in a gap. Moreover, it can be supposed that the strength of the link differs not only between mono- and bilingual persons but also varies among bilinguals. The language exposure hypothesis assumes that bilinguals with little language practice reveal a big gap and that persons with more practice show a small gap. However, there is scant empirical evidence for the effects of language exposure. Neither Oller et al.’s (2007) study on L1 and L2 nor in that of Gibson et al. (2012) on L1 was there any variation of the extent of the receptive–expressive gap dependent on language exposure. Only Gibson et al.’s (2014) study with proxy variables conformed to the hypothesis, revealing a larger gap among Spanish–English immigrant children with a lower level of language exposure. As the studies on the hypothesis produced contradictory results, the question of whether the language exposure hypothesis is in fact an adequate theory for explaining the gap remains unanswered.

### The Current Study

The aim of the present study was, first, to investigate central characteristics of the receptive–expressive gap in bilingual children’s L2 and to examine whether a receptive–expressive gap already exists at an early stage of language acquisition, i.e., at age of three and a half years. Secondly, we examined two central explanatory approaches to the receptive–expressive gap: the language familiarity hypothesis and the language exposure hypothesis. In order to test the language familiarity hypothesis we examined whether the differences in the size of the gap varies across groups of children with different L1s, respectively, groups of children with different language familiarities between the L1 and the German language (L2). To do this, we compared the size of the gap (in L2) in 11 groups of children with different first languages (e.g., Tamil–German, English–German). The language exposure hypothesis was tested with both proxy variables and variables of direct language contact. We postulated that children with higher linguistic input and more practice in the second language have a smaller gap in the second language. Accordingly, we expected that children with more contacts with German-speaking people—in the context of the family, with acquaintances, and in daycare—show a smaller discrepancy between language comprehension and language production.

## Materials and Methods

### Participants

The data for the present study stem from the first wave of the research project *Zweitsprache* [English translation: second Language] that was conducted in Basel, a city in the German-speaking part of Switzerland with an immigrant population of 34% (Bundesamt für Statistik [BFS], 2012a). The goals of the project were to investigate the second language development of immigrant preschoolers up to the first years of school and to analyze their childcare situation.

The sample consisted of 406 bilingual children (50.7% female) with German as a second language ranging in age between 34 and 53 months (*M* = 42.6, SD = 4.1). Due to limited number of immigrant children in Basel, the sample for the present analyses was collected over 4 years (August 2009, May 2010, May 2011, and May 2012) in four consecutive birth cohorts: 2005/06 (*n* = 59), 2006/07 (*n* = 90), 2007/08 (*n* = 117), and 2008/09 (*n* = 140).

The children originated from the following nations: Switzerland 29%, former Yugoslavia 11%, Turkey 10%, Sri Lanka 8%, Italy 8%, Portugal 4%, Great Britain 3%, Germany 3%, Spain 3%, the USA 2%, and a further 46 countries with a share of less than 2%. Eighty-one percent of the children, 10% of the mothers, and 16% of the fathers were born in Switzerland. The average period of residence of the parents born abroad was 10.2 years (SD = 7.0) among mothers and 13.6 years (SD = 8.3) among fathers. In 35% of the families German and the native language were used, and in 65% the native language was spoken mostly.

In regard to educational attainment, 24% of the mothers and 23% of the fathers reported that they had no post-compulsory qualification. Nineteen percent of the mothers and 23% of the fathers had completed vocational education; 18% of the mothers and 15% of the fathers had attended academic high school; and 39% of both mothers and fathers had attended a university of applied science or university. Parents without vocational training were overrepresented in comparison to the Swiss average (Bundesamt für Statistik [BFS], 2011). The average gross income was 79,300 Swiss Francs (SD = 34,400) and, in conformity with the expectations for families of immigrant background, it was below the national average of 112,400 Swiss Francs (Bundesamt für Statistik [BFS], 2012b).

### Measures

#### Receptive and Expressive Language Competence

Receptive and expressive German language competence (L2) were measured with the standardized language test *SETK-2* [Sprachentwicklungstest; English translation: Language Development Test] (Grimm, 2000). The test exists in a version for 2-year-old and a version for 3–5-year-old children. A pilot study revealed that most immigrant preschoolers are still at an initial stage of their second language acquisition (Keller, unpublished master’s thesis), and thus the version for younger children was chosen in order to reduce a floor effect and to display interindividual differences in the lower range of competence.

The SETK-2 consists of two subtests on receptive language (word comprehension, sentence comprehension) and two subtests on expressive language (word production, sentence production). The word comprehension tests and the sentence comprehension test are constructed similarly to the *Peabody Picture Vocabulary Test* (Dunn and Dunn, 2007). The child was presented with four colored pictures—a target item and three distractors, from which the corresponding picture had to be indicated. The instruction of the subtest on word comprehension ran: *Zeige mir den Hasen (example word)* [English translation: show me the bunny]; that of the subtest on sentence comprehension was: *Zeige mir das Bild: Der Vogel ist im Baum (example sentence)* [English translation: Show me the picture: the bird is in the tree]. In the two language production tests the child was shown colored pictures of objects (e.g., tree, swing) and situations (e.g., the horse is standing on the table) that had to be named in the subtest on word production and described in the subtest on sentence production. The instructions ran: *What is that?* and *What can you see here?* respectively. The evaluation of the subtest on word production was carried out in accordance with semantic criteria, that of the subtest on sentence production in accordance with both semantic and morpho-syntactic criteria.

The scales of the four subtests of the SETK-2 have a mean of 50 and a SD of 10 (*T*-values). As no standardized values exist for 3-year-old immigrant children with German as L2 the standard scores for the highest available age group (for 30–35-month-old monolingual children) were used. For receptive language competence the two subtests on word and sentence comprehension [*r*(404) = 0.71; *p*<0.001], and for expressive language those of the subtests on word and sentence production [*r*(404) = 0.86; *p*<0.001) were averaged. The reliabilities of the two receptive subtests are given with α = 0.84, those of the expressive subtests with α = 0.92. In order to create a measure for overall German language competence as a covariate, the *T*-values of the four subtests of the SETK-2 (Grimm, 2000) were averaged.

#### Language Groups

The grouping of the children according to their L1 was carried out on the basis of a parental questionnaire. The following language groups were formed: Albanian–German (*n* = 40), Bosnian/Serbian/Croatian–German (*n* = 26), English–German (*n* = 32), French–German (*n* = 19), Italian–German (*n* = 38), Kurdish–German (*n* = 13), Portuguese–German (*n* = 24), Spanish–German (*n* = 36), Tamil–German (*n* = 42), Turkish–German (*n* = 65), and a mixed group with rare first languages^1^ (*n* = 71). Forty-five parents stated more than one L1. In this case the grouping was carried out on the basis of the first named language.

#### Language Contact

In accordance with Gibson et al. (2012) the following variables derived from the parental questionnaire were used as proxies for language contact: country of birth (1 = foreign countries, 2 = Switzerland), German-speaking daycare attendance (1 = no, 2 = yes), duration of German language exposure (1 = less than a year, 2 = 1 year or more), mother’s length of Swiss residency (1 = 8 years or less, 2 = more than 8 years), mother’s German proficiency (1 = low, 2 = high), mother’s education (1 = no academic high school, 2 = academic high school), number of adults at home (1 = parents and others, 2 = only parents), number of children at home (1 = one or two children, 2 = more than two children), birth order (1 = first- or only-born, 2 = later-born). The first-named manifestations of the proxies indicate a slight contact and the second manifestations a greater degree of contact with the German language (see Gibson et al., 2012).

As direct language contact variables the parental questionnaire information on use of German in the family, time spent in German-speaking daycare, and contact with German-language speakers outside the family were used. (a) The *use of German in the family* was assessed with the question: does your family speak predominantly German at home or another language? On a 5-point scale the parents indicated whether 1 = exclusively German, 2 = mostly German, 3 = both languages equally, 4 = mostly another language or 5 = exclusively another language (reversed item) was spoken (Keller and Grob, 2013). (b) The *amount of time spent in German-speaking daycare* was measured in hours per week. The additional naming of the institution served the purpose of checking whether it was a German-speaking institution. (c) The *frequency of contacts with German-speaking children and German-speaking adults* was ascertained in each case with a mean value on the basis of a 4-point scale with the manifestations 1 = rarely to 4 = daily. The interval- and ordinal-scaled proxy and language contact variables were dichotomized through a median split in analogy to the study of Gibson et al. (2012).

### Procedure

The recruitment for the sample was carried out on the basis of official information from the Registration Office of the City of Basel for the years 2009–2012. Families throughout the entire area with a pre-kindergarten child were sent a language development questionnaire (DaZ-E; Keller and Grob, 2013) and a written request to participate in the research study 18 months before obligatory entry into kindergarten^2^. The documents and the questionnaire were made available to the parents in Albanian, Bosnian/Serbian/Croatian, English, French, German, Italian, Portuguese, Spanish, Tamil, and Turkish. Among families who had given their written informed consent to participation in the study, the sample was selected on the basis of gender, age distribution, and representativity of nationalities.

Language tests were conducted by trained native German-speaking research assistants with a Bachelor of Science degree in psychology. The tests took place in the homes of the families and had a duration of about 30 min. If the parents had only slight knowledge of German and the research assistant did not have the corresponding language competence, an intercultural intermediary of the Cantonal Ministry of Education was called in to explain the test procedure to the parents. The study was approved by the Ethic Review Committee of the City and the County of Basel (EKBB).

## Results

### Characteristics of the Receptive–Expressive Gap

Our first research question explored the characteristics of the receptive–expressive gap. The means of receptive and the expressive competence were significantly below the mean of the norm sample [one-sample *t*-tests: receptive: *t*(405) = -13.32, *p* < 0.001; expressive: *t*(405) = -35.54, *p* < 0.001]. Receptive language competence was higher compared with expressive language competence. As hypothesized, there was a significant receptive–expressive gap in favor of language comprehension [paired *t*-test: *t*(405) = 25.38, *p* < 0.001]. The effect size expressed in Cohen’s *d* = 1.26 can be interpreted as being very large (Cohen, 1988). A total of 90.1% of the children revealed a better receptive competence in comparison to their expressive competence; the expressive competence was better developed among only 6.2% of the bilingual children. The share of children with a receptive–expressive gap in favor of receptive language according to Gibson et al.’s (2014) definition amounted to 62.3%, whereas only four children (1%) showed a significant gap in the opposite direction.

### Language Familiarity Hypothesis

In order to test the language familiarity hypothesis the differences in the receptive–expressive gap (in L2) were compared among eleven groups of children with different L1s. As can be seen from **Table 1** a gap in favor of receptive language competence emerged in all language groups. The size of the gap was between Cohen’s *d* = 0.99 (for children with Turkish as their L1) and *d* = 1.68 (for children with Albanian as their L1), thus all were in a high effect size range (Cohen, 1988). Comparisons using *t*-tests for paired samples revealed a significant receptive–expressive gap in each of the 11 language groups after Bonferroni correction for multiple testing (paired *t*-test, all *p*s < 0.0045). To test the language familiarity hypothesis a two-way covariance analysis (ANCOVA) with the within-subject factor language modality (M: receptive, expressive) was carried out. The 11 language groups (L: Albanian–German, Bosnian/Serbian/Croatian–German, English–German, French–German, Italian–German, Kurdish–German, Portuguese–German, Spanish–German, Tamil–German, Turkish–German, and the mixed group of rare first languages) were used as between-subjects factor. Covariates were gender and overall German language level. However, due to the non-significant effect the covariate gender was excluded from analyses testing the causes of the gap [*F*(1,392) = 0.023, *p* = 0.880]. The results revealed a significant main effect for the factor modality (M), *F*[1,394) = 8.471, *p* < 0.01, $\eta_{p}^{2}$ = 0.021] and a non-significant main effect for the factor language group (L), *F*(10,394) = 1.738, *p* = 0.070, $\eta_{p}^{2}$ = 0.042. The interaction effect M × L, which is of interest for the language familiarity hypothesis, was not significant, *F*(10,394) = 1.562, *p* = 0.116, $\eta_{p}^{2}$ = 0.038.

**Table 1:** Means and SD on receptive and expressive German language competence (L2) as a function of L1 (*T*-scores).

		Rec (L2)		Exp (L2)		Gap (L2)	
L1s	*n*	*M*	SD	*M*	SD	Diff	*d*
Albanian	40	39.21	10.97	27.98	6.51	11.23	1.68
BSK	26	42.06	11.79	30.88	8.74	11.18	1.49
English	32	45.22	15.43	32.48	12.76	12.74	1.13
French	19	50.37	10.96	39.47	14.07	10.90	1.25
Italian	38	44.33	11.98	33.01	10.72	11.32	1.46
Kurdish	13	37.08	9.36	30.70	6.26	6.38	1.12
Portuguese	24	41.73	13.46	31.27	8.90	10.46	1.21
Spanish	36	44.72	10.90	33.44	9.78	11.28	1.55
Tamil	42	38.99	10.94	28.98	6.69	10.01	1.33
Turkish	65	39.20	12.04	31.14	11.09	8.06	0.99
Mixed	71	41.73	12.66	32.85	11.47	8.88	1.20
Total	406	41.85	12.32	31.75	10.34	10.10	1.26

*Rec, Receptive Language (SETK-2); Exp, Expressive Language (SETK-2). Diff, Difference score between Receptive and Expressive Language. BSK, Bosnian/Serbian/Croatian.*

**FIGURE 1:**
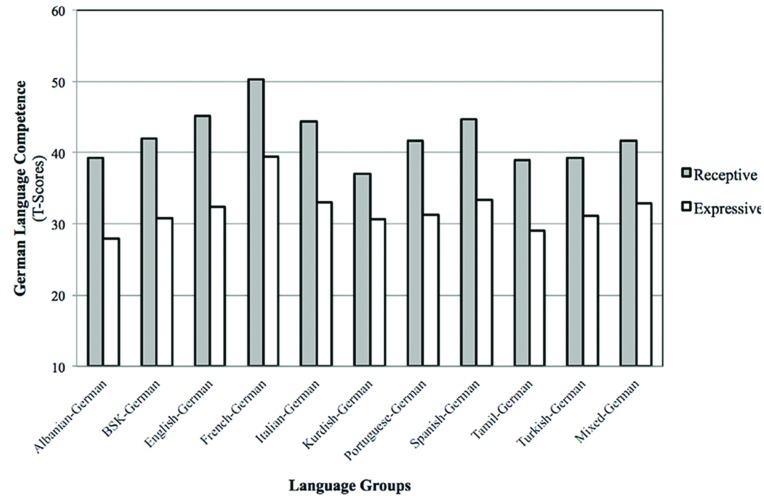
**Means on receptive and expressive German language competence (L2); BSK, Bosnian/Serbian/Croatian**.

Results show that a significant receptive–expressive gap exists in all 11 language groups (Albanian–German, English–German, French–German, Turkish–German, etc.), but that the size of the gap does not vary significantly across the different language groups (**Figure 1**). Hence the data do not support the language familiarity hypothesis and it thus can be concluded that the gap does not depend on the language familiarity of L1 and L2. As there were no significant gap differences it was not necessary to sort the language groups in order to language familiarity of L1 and L2.

### Language Exposure Hypothesis

The language exposure hypothesis was tested, on the basis of proxy variables and on the basis of direct language contact variables for German as L2. In analogy to the study by Gibson et al. (2012) a separate two-way ANCOVA was calculated for each proxy and language contact variable. The within-subject factor was modality (M: receptive, expressive), the between-subject factor was the dichotomous variable language exposure (E: low, high), and as covariate the overall German language level was used. The language exposure hypothesis was judged on the basis of the interaction terms M × E. Because directional hypotheses were formulated, the two-way *p*-values in **Tables 2** and **3** were halved (Bortz, 2005). As can be seen from the interactions (M × E) in **Table 2** none of the eight proxy variables studied explained variance in the gap in German. In line with Gibson et al.’s (2012) study, our analyses with proxy variables revealed no differences in the size of the gap. This means that children who on the basis of the proxy variables had had more frequent language exposure and children for whom a lower amount of language exposure was assumed showed similar gap sizes.

**Table 2:** Two-way analyses of covariance in German language competence by proxy of language contact and language modality.

		*n*	Rec (L2)		Exp (L2)		Diff	df	*F*_M × E_	*p*	$\eta_{p}^{2}$
Proxy variables			*M*	SD	*M*	SD					
Country of birth	Foreign countries	76	41.53	12.22	31.07	8.16	10.46	1, 391	0.222	0.638	0.001
	Switzerland	318	41.85	12.23	31.83	10.38	10.02				
Daycare	No	121	41.66	10.12	31.49	7.39	10.17	1, 397	0.085	0.770	0.000
	Yes	279	41.91	12.46	32.00	10.94	9.91				
Exposure of L2	<1 year	188	41.73	10.32	31.41	7.50	10.32	1, 382	0.565	0.453	0.001
	≥1 year	197	42.25	11.91	32.58	11.12	9.67				
Mother’s length of Swiss residency	≤8 years	202	41.50	12.43	31.81	7.97	9.69	1, 391	1.434	0.232	0.004
	>8 years	192	42.10	12.15	31.55	10.18	10.55				
Mother’s German proficiency	Low	253	41.15	11.43	31.41	8.57	9.74	1, 391	0.490	0.484	0.001
	High	141	42.76	12.32	32.45	12.21	10.31				
Mother’s education	No high school	147	41.89	11.53	31.49	9.06	10.40	1, 338	0.278	0.599	0.001
	High school	194	42.75	12.98	32.78	11.68	9.97				
Adults at home^a^	Parents and others	28	41.72	12.07	31.78	8.81	9.94	1, 393	0.966	0.326	0.002
	Only parents	368	42.46	12.40	31.04	10.33	11.42				
Children at home^a^	≤2 children	328	41.73	12.19	31.70	10.11	10.03	1, 403	0.123	0.726	0.000
	>2 children	78	42.36	12.91	32.00	11.36	10.36				
Birth order	First-born	220	42.03	13.14	32.41	11.14	9.62	1, 379	2.407	0.122	0.006
	Later-born	162	41.93	11.44	31.13	9.44	10.8				

*Means, F-scores, p-values, and $\eta_{p}^{2}$ adjusted for overall German language score. F_M × E_, F-Score of the interaction term modality × proxy of language contact. Rec, Receptive Language; Exp, Expressive Language. ^a^The results of the variables Adults at home and Children at home must be interpreted with caution on account of highly unequal group sizes.*

**Table 3:** Two-way analysis of covariance in German language competence by language contact and language modality.

		*n*	Rec (L2)		Exp (L2)		Diff	df	*F*_M × E_	*p*	$\eta_{p}^{2}$
Contact variables			*M*	SD	*M*	SD					
German in family	Native language	276	41.65	11.94	31.13	8.71	10.52	1, 402	3.174	0.076	0.008
	Both language	129	42.15	12.72	33.05	12.77	9.10				
German in daycare	≤12 h/week	243	42.06	11.78	31.46	8.56	10.60	1, 393	3.670	0.056	0.009
	>12 h/week	153	41.67	12.20	32.59	11.88	9.08				
German with others	Less than daily	222	41.79	11.27	31.11	7.89	10.68	1, 377	4.988	0.026	0.013
	Daily	158	42.06	12.62	33.19	12.22	8.87				

*Means, F-scores, p-values, and $\eta_{p}^{2}$ adjusted for overall language score. F_M × E_, F-Score of the interaction term modality × exposure. Rec, Receptive Language; Exp, Expressive Language.*

In a next step, the language exposure hypothesis was tested with direct language contact variables. In analogy to the analyses with proxy variables, a two-way ANCOVA was calculated for each language contact variable with repeated measurements in the factor modality (M: receptive, expressive). As between-subject factors the three direct language contact variables (a) *German use in family*, (b) *German use in daycare*, and (c) *German use with others* (E: low, high) were used (**Table 3**). In the first analysis on *German use in family* there was a significant interaction (M × E), *F*(1,402) = 3.174, *p*_one-tailed_ = 0.038, $\eta_{p}^{2}$ = 0.008. Children from families which used L2, German, alongside their native language revealed a lower difference between receptive and expressive language in comparison to children from homes in which the native language was exclusively or predominantly spoken. The interaction term of the second analysis on *German use in daycare* was significant, *F*(1,393) = 3.670, *p*_one-tailed_ = 0.028, $\eta_{p}^{2}$ = 0.009. Children who attended a daycare institution for more than 12 h a week showed a lower receptive–expressive gap in comparison with their peers who were exclusively looked after in the family or were in daycare for fewer than 12 h. In the third analysis on *German use with others* there was, again as assumed, a significant interaction between language contact and modality, *F*(1,377) = 4.988, *p*_one-tailed_ = 0.013, $\eta_{p}^{2}$ = 0.013. For children who had contact on a regular base with German-speaking children and adults among their relations, acquaintances and neighbors the gap was less strongly pronounced than for children with fewer contacts with German-speaking persons. Even though the effect sizes are small (Cohen, 1988), the data on direct language contact provide evidence for the language exposure hypothesis.

## Discussion

The present study had two main purposes. First, it aimed to examine the features of the receptive–expressive gap in bilingual children’s L2. Second, it investigated the causes of the receptive–expressive gap focusing on two approaches—the language familiarity and the language exposure hypotheses.

### Characteristics of the Expressive–Receptive Gap

The results on the prevalence of the gap make it clear that the bilingual gap is a normative phenomenon that already exists in the early stage of second language acquisition. The great majority of the bilingual children (90.1%) revealed a greater receptive than expressive competence and 62.3% of the cases showed a receptive–expressive gap according to Gibson et al.’s (2014) definition. Moreover, the current study displayed that the receptive–expressive gap amounted to 1 SD in preschoolers’ L2.

Thus, our study extends the knowledge of a gap in L2 among kindergarten children and primary school students (Windsor and Kohnert, 2004; Oller et al., 2007; Swanson et al., 2008; Sheng et al., 2011) showing that receptive–expressive gap already exists in the early stage of second language acquisition. If one focuses on the earliest phase in second language acquisition, parallels between the *silent period* (Gibbons, 1985) and the bilingual receptive–expressive gap can be recognized. The silent period is one of the early stages of second language acquisition, when children have already realized that the use of L1 in the context of L2 is not expedient. In this phase the children do not express themselves orally but they observe and actively listen. The silent period is possibly the first phase in the development of the receptive–expressive gap, which indicates that second language production has not yet started but a basic language comprehension already exists or is in the process of developing. In this sense the silent period could be considered to be a preliminary stage in the emergence of the receptive–expressive gap.

It was surprising, however, that the gap in L2 amounted to 1 SD and was thus clearly larger than was to be expected on the basis of the majority of previous studies (e.g., Windsor and Kohnert, 2004; Kan and Kohnert, 2005; Barnett et al., 2007; Oller et al., 2007; Lesaux et al., 2010; Sheng et al., 2011; Gibson et al., 2014). According to the existing literature a gap of maximum one-half a SD was assumed among bilingual children in L2 (see Gibson et al., 2012). The discrepancy between the results of our study and previous analyses might be traced back to different amounts of language contact across studies. The current results indicate that contextual conditions or the frequency of the use of the language at the given moment contribute to the size of the gap in bilinguals. For the three and a half year old children in our sample, who are primarily cared for by parents, the local language (L2, German) was still very unfamiliar, in contrast to the situation in a large part of the samples in previous studies.

### Causes of the Gap

As is shown by the analyses on the language familiarity hypothesis, the gap in L2 is a robust phenomenon over and beyond L1s used. There was a significant gap in all 11 language groups. The results supplement the findings of Oller et al. (2007) and Gibson et al. (2012), which have already demonstrated a pronounced robustness of the gap in relation to socio-demographic features and different teaching methods. No empirical evidence could be found for the language familiarity hypothesis, according to which different gap sizes are expected in the eleven language groups. It can therefore be assumed that the receptive–expressive gap cannot be traced back to the linguistic familiarity of L1 and L2. Differential transfer effects from L1 to L2 specifically related to modality must consequently be regarded as improbable—at least in early language acquisition.

In contrast to the language familiarity hypothesis, our data provide evidence for the language exposure hypothesis. Bilingual children with more opportunities for contact and interaction with German-speaking persons reveal a smaller gap. This is demonstrated in the use of German in the family, in contacts with German-speaking persons outside the family context as well as in childcare settings. Even though the effect size is small, the language contact does, consequently, provide a possible cause of the rise of a discrepancy between language comprehension and language production. A smaller gap occurred especially when regular contact with speakers of the local language lasting several hours per week existed. This result is in line with Gibson et al.’s (2014) study, which showed that the amount of language exposure is related to the size of the gap in bilingual children.

The results of the present study might be related to the weaker link hypothesis as proposed by Gollan et al. (2002). Just as differences in lexical access were detected between mono- and bilingual persons (Gollan and Acenas, 2004; Yan and Nicoladis, 2009), it appears that gap differences exist among bilinguals depending on language practice. Possibly the big gap in children with little language contact is attributable to a weakly pronounced link between the semantic and the phonological system. According to the weaker links hypothesis, a weak connection hinders lexical access but has no or little implications on language comprehension, which results in a receptive–expressive gap.

However, in the present study exposure to L2 was operationalized through interactional opportunities in L2, rated by parental report. We did not use direct observational measures to assess in what way these opportunities were taken. Therefore, in the present study it cannot be differentiated as to whether passive contact with the German language or active participation in communication is associated with a smaller gap. To ascertain the underlying processes responsible for the receptive–expressive gap, further research is needed.

In analogy to Gibson et al.’s (2012) study, in which the language exposure hypothesis was tested for L1 on the basis of proxy variables for language contact without finding any effects, our study failed to explain a difference in the gap in L2 using the same proxy variables. This result reflects the fact that socio-demographic features are insufficiently associated with certain language practices of families and accordingly provide rather unfavorable proxy variables for language exposure.

### Strengths, Limitations, and Future Directions

A major strength of this study is the heterogeneous composition of the sample, which makes it possible for the first time to test the language familiarity hypothesis empirically. The sample with 11 different language groups from both Indo-European and non Indo-European language families presented an ideal starting point for testing the language familiarity hypothesis and extends our knowledge of the causes of the gap (Oller et al., 2007; Gibson et al., 2012, 2014). A further strength is that the present findings supplement previous studies examining the receptive–expressive gap in early second language acquisition. The finding of a significant receptive–expressive gap is of particular relevance for language diagnostics, showing the need for assessing both receptive and expressive language competence for a complete understanding of the language development in bilingual children. A third strength to be emphasized is the use of a language development test consisting of different subtests. These not only refer to receptive and expressive vocabulary, but also include the comprehension and production of entire sentences. Although in previous studies on preschool children the focus was frequently on vocabulary as the most salient and most easily graspable characteristic (Kan and Kohnert, 2005; Sheng et al., 2011), it is precisely the embedding of the individual words in a sentence structure that is central for everyday communication.

For future research it would be desirable to conceive studies with a longitudinal design that take both L1 and L2 into account. This would enable us to trace changes in the gap in both languages and to relate them to one another. In addition, it is necessary to examine whether the same explanatory mechanisms underlie the receptive–expressive gap in both L1 and L2.

## Conclusion

In sum, the current study extends our knowledge of bilingual language development and draws attention to a distinguishing characteristic between bilingual and monolingual language development, hitherto neglected in developmental psychology. The results showed that the receptive–expressive gap is a normative phenomenon in bilingual preschoolers. The results on the prevalence of the gap are relevant especially in regard to the clinical and developmental diagnostics of bilingual children. The long-standing call for specific bilingual language tests or at least for bilingual norms acquires more weight as a result of our study (Roberts et al., 1999; Castro et al., 2011). Tests for bilingual children could help to avoid the mistaken classification of children with a normative bilingual language profile as clinically conspicuous. In language diagnostics it must also be taken into account that a restriction to one modality can, in view of the great discrepancy, lead to an under- or overestimation of the general second language competence. The analyses of the causes of the gap revealed that it is not the relative familiarity of the languages but rather language exposure to L2 that explains individual differences in the size of the gap. These findings are of central significance not only from the perspective of basic research but also for practical work with bilingual children.

## Conflict of Interest Statement

The authors declare that the research was conducted in the absence of any commercial or financial relationships that could be construed as a potential conflict of interest.

## Footnotes

1 First languages with a frequency of less than ten children per language in the present sample: Arabic, Armenian, Bengali, Berber, Bulgarian, Chechen, Chinese, Czech, Danish, Dutch, Finnish, Galician, Hebrew, Hindi, Hungarian, Japanese, Kyrgyz, Luxembourgish, Malayalam, Norwegian, Pakistani, Persian, Polish, Punjabi, Romansh, Russian, Shona, Singhalese, Slovenian, Swedish, Telugu, Thai, Tigrinya, Ukrainian, Urdu, Vietnamese.

2 Entry into kindergarten takes place in Basel on completion of the fourth year of life.

## References

[B1] Barac R. Bialystok E. Castro D. C. Sanchez M. (2014). The cognitive development of young dual language learners: A critical revew. *Early Child. Res. Q.* 29 699–714. 10.1016/j.ecresq.2014.02.003 25284958 PMC4180217

[B2] Barnett W. S. Yarosz D. J. Thomas J. Jung K. Blanco D. (2007). Two-way and monolingual English immersion in preschool education: an experimental comparison. *Early Child. Res. Q.* 22 277–293. 10.1016/j.ecresq.2007.03.003

[B3] Bialystok E. (2015). Bilingualism and the development of executive function: the role of attention. *Child Dev. Perspect.* 9 117–121. 10.1111/cdep.12116 26019718 PMC4442091

[B4] Bortz J. (2005). *Statistik für Human- und Sozialwissenschaftler [Statistics for Social Scientists]*, 6th Edn Berlin: Springer Medizin Verlag Heidelberg.

[B5] Brito N. H. Grenell A. Barr R. (2014). Specificity of the bilingual advantage for memory: examining cued recall, generalization, and working memory in monolingual, bilingual, and trilingual toddlers. *Front. Psychol.* 5:1369 10.3389/fpsyg.2014.01369 PMC4251311 25520686

[B6] Bundesamt für Statistik [BFS]. (2011). *Bildung, Wissenschaft [Education, Science]*. Available at: http://www.bfs.admin.ch/bfs/portal/de/index/themen/15.html

[B7] Bundesamt für Statistik [BFS]. (2012a). *Bestand der Ständigen Ausländischen Wohnbevölkerung nach Wohnkanton und Ausländergruppe Ende August 2012 [Stability of Permanent Immigrant population Listed by Canton of Residence and Immigrant Group at the end of August 2012]*. Available at: http://www.bfm.admin.ch/content/dam/data/migration/statistik/auslaenderstatistik/aktuelle/ausl-nach-kanton/107-bevoelkerung-kt-2012-08-d.pdf

[B8] Bundesamt für Statistik [BFS]. (2012b). *Löhne Erwerbseinkommen—Detaillierte Daten [Wages, income—Detailed data]*. Available at: http://www.bfs.admin.ch/bfs/portal/de/index/themen/03/04/blank/data/00.html

[B9] Castro D. C. Páez M. M. Dickinson D. K. Frede E. (2011). Promoting language and literacy in young dual language learners: research, practice, and policy. *Child Dev. Perspect.* 5 15–21. 10.1111/j.1750-8606.2010.00142.x

[B10] Chen S. H. Zhou Q. Uchikoshi Y. Bunge S. A. (2014). Variations on the bilingual advantage? Links of Chinese and English proficiency to Chinese American children’s self-regulation. *Front. Psychol.* 5:1069 10.3389/fpsyg.2014.01069 PMC4179764 25324795

[B11] Cohen J. (1988). *Statistical Power Analysis for the Behavioral Sciences*, 2nd Edn Hillsdale: Lawrence Erlbaum Associates.

[B12] Cummins J. (1979). Linguistic interdependence and the educational development of bilingual children. *Rev. Educ. Res.* 49 222–251. 10.3102/00346543049002222

[B13] Dunn L. M. Dunn D. M. (2007). *Peabody Picture Vocabulary Test*, 4th Edn Minneapolis, MN: Wascana.

[B14] Farnia F. Geva E. (2011). Cognitive correlates of vocabulary growth in English language learners. *Appl. Psycholinguist.* 32 711–138. 10.1017/S0142716411000038

[B15] Filippi R. Morris J. Richardson F. M. Bright P. Thomas M. S. C. Karmiloff-Smith A. (2015). Bilingual children show an advantage in controlling verbal interference during spoken language comprehension. *Biling. (Camb. Engl.)* 18 490–501. 10.1017/S1366728914000686 26146479 PMC4486347

[B16] Genesee F. Geva E. (2006). “Cross-linguistic relationships in working memory, phonological processes, and oral language,” in *Developing Literacy in Second-Language Learners: Report of the National Literacy Panel on Language-Minority Children and Youth*, eds August D. Shanahan T. (Mahwah, NJ: Lawrence Erlbaum Associates), 175–183.

[B17] Gibbons J. (1985). The silent period: An examination. *Lang. Learn.* 35 255–267. 10.1111/j.1467-1770.1985.tb01027.x

[B18] Gibson T. Oller D. Jarmulowicz L. Ethington C. (2012). The receptive–expressive gap in the vocabulary of young second-language learners: Robustness and possible mechanisms. *Biling. (Camb. Engl.)* 15 102–116. 10.1017/S1366728910000490 22247648 PMC3254083

[B19] Gibson T. A. Peña E. D. Bedore L. M. (2014). The relation between language experience and receptive–expressive semantic gaps in bilingual children. *Int. J. Biling. Educ. Biling.* 17 90–110. 10.1080/13670050.2012.743960 PMC5902174 29670456

[B20] Gollan T. H. Acenas L.-A. R. (2004). What is a TOT? Cognate and translation effects on tip-of-the-tongue states in Spanish–English and Tagalog–English bilinguals. *J. Exp. Psychol. Learn. Mem. Cogn.* 30 246–269. 10.1037/0278-7393.30.1.246 14736310

[B21] Gollan T. H. Montoya R. I. Werner G. A. (2002). Semantic and letter fluency in Spanish–English bilinguals. *Neuropsychology* 16 562–576. 10.1037/0894-4105.16.4.562 12382994

[B22] Grimm H. (2000). *Sprachentwicklungstest für Zweijährige Kinder (SETK-2) [Language Development Test for Two-Year-Old Children (SETK-2)]*. Göttingen: Hogrefe.

[B23] Grüter T. (2005). Comprehension and production of French object clitics by child second language learners and children with specific language impairment. *Appl. Psycholinguist.* 26 363–391. 10.1017/S0142716405050216

[B24] Hemsley G. Holm A. Dodd B. (2010). Patterns in diversity: lexical learning in Samoan–English bilingual children. *Int. J. Speech Lang. Pathol.* 12 362–374. 10.3109/17549501003721064 20441411

[B25] Hoff E. (2013). Interpreting the early language trajectories of children from low-SES and language minority homes: implications for closing achievement gaps. *Dev. Psychol.* 49 4–14. 10.1037/a0027238 22329382 PMC4061698

[B26] Hutchinson J. M. Whiteley H. E. Smith C. D. Connors L. (2003). The developmental progression of comprehension-related skills in children learning EAL. *J. Res. Read.* 26 19–32. 10.1111/1467-9817.261003

[B27] Kan P. F. Kohnert K. (2005). Preschoolers learning Hmong and English: lexical-semantic skills in L1 and L2. *J. Speech Lang. Hear. Res.* 48 372–383. 10.1044/1092-4388(2005/026) 15989399

[B28] Kang J. Y. (2012). Do bilingual children possess better phonological awareness? Investigation of Korean monolingual and Korean-English bilingual children. *Read. Writ.* 25 411–431.

[B29] Keller K. Grob A. (2013). Elternfragebogen zu den Deutschkenntnissen mehrsprachiger Kinder [Parent questionnaire to the German language skills of bilingual children]. *Z. Pädagog. Psychol.* 27 169–180. 10.1024/1010-0652/a000102

[B30] Lesaux N. Crosson A. Kieffer M. Pierce M. (2010). Uneven profiles: Language minority learners’ word reading, vocabulary, and reading comprehension skills. *J. Appl. Dev. Psychol.* 31 475–483. 10.1016/j.appdev.2010.09.004 21243117 PMC3020082

[B31] Oller D. K. Jarmulowicz L. (2007). “Language and literacy in bilingual children in the early school years,” in *Blackwell Handbook of Language Development*, eds Hoff E. Shatz M. (Oxford: Blackwell), 368–386.

[B32] Oller D. K. Pearson B. Z. Cobo-Lewis A. B. (2007). Profile effects in early bilingual language and literacy. *Appl. Psycholinguist.* 28 191–230. 10.1017/S0142716407070117 22639477 PMC3358777

[B33] Pearson B. Z. Fernández S. C. Oller D. K. (1993). Lexical development in bilingual infants and toddlers: comparison to monolingual norms. *Lang. Learn.* 43 93–120. 10.1111/j.1467-1770.1993.tb00174.x

[B34] Roberts J. E. Burchinal M. Durham M. (1999). Parents’ report of vocabulary and grammatical development of African American preschoolers: child and environmental associations. *Child Dev.* 70 92–106. 10.1111/1467-8624.00008 10191517

[B35] Sachse S. Budde N. Rinker T. Groth K. (2010). Mehrsprachige kinder in vorschulischen sprachfördermassnahmen. Soziodemografischer hintergrund und sprachleistungen [Multilingual children in preschool language learning programs]. *Logos Interdisziplinär* 18 337–345.

[B36] Sebastian-Galles N. Albareda-Castellot B. Weikum W. Werker J. (2012). A bilingual advantage in visual language discrimination in infancy. *Psychol. Sci.* 23 994–999. 10.1177/0956797612436817 22810164

[B37] Sheng L. Lu Y. Kan P. (2011). Lexical development in Mandarin–English bilingual children. *Biling. Lang. Cogn.* 14 579–587. 10.1017/S1366728910000647

[B38] Swanson H. Rosston K. Gerber M. Solari E. (2008). Influence of oral language and phonological awareness on children’s bilingual reading. *J. Sch. Psychol.* 46 413–429. 10.1016/j.jsp.2007.07.002 19083366

[B39] Verhoeven L. (2000). Components in early second language reading and spelling. *Sci. Stud. Read.* 4 313–330. 10.1207/S1532799XSSR0404_4

[B40] Windsor J. Kohnert K. (2004). The search for common ground: Part I. Lexical performance by linguistically diverse learners. *J. Speech Lang. Hear. Res.* 47 877–890. 10.1044/1092-4388%282004/065%29 15324292

[B41] Yan S. Nicoladis E. (2009). Finding le mot juste: differences between bilingual and monolingual children’s lexical access in comprehension and production. *Biling. Lang. Cogn.* 12 323–335. 10.1017/S1366728909990101

